# Osteotomy in direct sinus lift. A comparative study of the rotary technique and ultrasound

**DOI:** 10.4317/medoral.17599

**Published:** 2011-12-06

**Authors:** María Peñarrocha-Diago, Miguel Peñarrocha-Diago, Cristina Sanchez-Recio, David Peñarrocha-Oltra, Javier Romero-Millán

**Affiliations:** 1 DDS, PhD Associate Professor of Oral Surgery, Valencia University Medical and Dental School; 2 DDS, MD, PhD Chairman of Oral Surgery and Director of the Master in Oral Surgery and Implantology, Valencia University Medical and Dental School; 3 PhD Master in Oral Surgery and Implantology, Valencia University Medical and Dental School; 4 DDS, DDS Master ´s Degree Student of Oral Surgery and Implantology, Valencia University medical and Dental School, Valencia

## Abstract

Purpose: The present study investigates sinus membrane rupture in direct maxillary sinus lift with the rotary technique and with ultrasound, examining the survival of implants placed after sinus augmentation, and analyzing the bone gain obtained after the operation and 12 months after placement of the prosthetic restoration. Material and Methods: A retrospective study was made of 45 patients requiring maxillary sinus lift or augmentation for implant-prosthetic rehabilitation. Use was made of the hand piece and ostectomy drills for the rotary technique, and of specific tips for ultrasound. The implant success criteria were based on those developed by Buser. The bone gain obtained as a result of sinus lift was calculated from the postoperative panoramic X-rays. Results: A total of 57 direct elevations of the maxillary sinus were carried out: 32 with the rotary technique and 25 with ultrasound. Perforations of Schneider’s membrane with the rotary technique and ultrasound occurred in 7% and 1.7% of the cases, respectively, with membrane integrity being preserved in 91.2%. Of the 100 implants placed, 5 failed after one year of follow-up in the rotary technique group, while one implant failed in the ultrasound group. The rotary technique in turn afforded a bone gain of 5.9 mm, versus 6.7 mm with ultrasound. Conclusions: Perforations of the membrane sinusal in direct lift were more frequent with the rotary technique (7%) than with ultrasound (1.7%). Implant survival and bone gain were both greater when ultrasound was used.

** Key words:**Bone sectioning, maxillary sinus augmentation, piezosurgery.

## Introduction

Sinus elevation allows maxillary bone augmentation and thus facilitates implant rehabilitation in patients with severe posterior maxillary atrophy. In direct maxillary sinus lift a vestibular osteotomy is performed, a bone window is prepared, and access is gained to the maxillary sinus for elevation of the membrane. The bone perforation can be carried out using an osteotomy drill in the context of the conventional rotary technique, or using ultrasound tips (1).

In elevation with the rotary technique, the main intraoperative complication is perforation of Schneider’s membrane, which is observed in between 10-35% of all such operations (2), and which usually occurs in the osteotomy drilling phase while preparing the window for access to the sinus (3). With the purpose of reducing the risk of perforating Schneider’s membrane, vestibule osteotomy using ultrasound has been proposed, as this reduces the risk of soft tissue damage (4) and percentage membrane perforation to 7% (5). Some studies in the literature are preliminary descriptions of the technique (6), while others present isolated cases (7), and others in turn report case series - no significant differences being observed between the two techniques (8).

The present retrospective study was designed to compare the performance of the rotary technique versus ultrasound in application to sinus lift, analyzing sinus membrane rupture in direct maxillary sinus lift with both instruments, examining the post-augmentation survival of implants 12 months after prosthetic restoration, and analyzing the bone gain obtained after the operation and 12 months after placement of the prosthetic restoration.

## Material and Methods

Material

A retrospective study was made of all successive patients subjected to direct maxillary sinus lift between July 2003 and April 2008. In the patients subjected to sinus lift between July 2003 and April 2005, use was made of the rotary technique, while ultrasound was used in those operated upon between May 2005 and April 2008. The study included 48 patients subjected to direct maxillary sinus lift with either the rotary technique (24 patients) or ultrasound (24 patients).

The following inclusion criteria were established: 1.- Partial or total maxillary edentulism, with a residual alveolar crest height of ≤ 6 mm; and 2.- Implant based rehabilitation using direct sinus lift with the rotary technique or with ultrasound.

Patients in whom the study protocol proved incomplete were excluded from the study, and a follow-up of at least one year after loading was required.

Methods

All the operations were carried out by the same surgeon. In direct maxillary sinus lift with the rotary technique, use was made of a hand piece and rounded tungsten carbide drill with abundant sterile physiological saline irrigation. In turn, in direct maxillary sinus lift with ultrasound (Surgysonic®, Esacrom, Imola, Italy), use was made of Surgysonic® ostectomy ultrasound tips (ES015 and ES015A) likewise with abundant sterile physiological saline irrigation.

In the rotary technique, Schneider’s membrane was detached and raised with special University of Loma Linda curettes (Hu-Friedy®, Rotterdam, Holland), while in the ultrasound technique use was made of the ES004 and ES003B tips. The sinus membrane was suspected after raising and classified as either intact or perforated.

Grafting was carried out using particulate autologous bone, bovine heterologous bone (Bio-Oss®, Geistlich Pharma AG. Switzerland), or a 50% mixture of both. In the ultrasound technique, the autologous bone was harvested from the retromolar trigone or maxillary tuberosity with the saw-shaped ES007 tip and the ES003 bone particle collecting tip.

For preparation of the implant beds we combined the rotary instrumentation with osteotomes. After preparing the beds, the implants were placed, the space achieved as a result of sinus lift was filled with autologous bone particles, heterologous bone or a mixture of both, and covering was carried out with a reabsorb able collagen membrane adapted to the cavity (Lyostypt®, B. Braun, Aesculap, Tuttlingen, Germany). Based on the amount of remaining bone at the time of sinus lift, the implants were placed in the same surgical step if the alveolar crest height exceeded 4 mm, while the implants were placed between 4-6 months later in second step surgery in those patients presenting crest heights of less than 4 mm.

Defcon® (Impladent; Senmenat, Barcelona, Spain) and Straumann® implants (Institut Straumann; Base, Switzerland) were placed, exposed or submerged, depending on whether the patients wore removable dentures or not.

The number of failed implants was recorded, and implant success was rated according to the criteria developed by Buser in 1990 (9).

**Figure 1:**
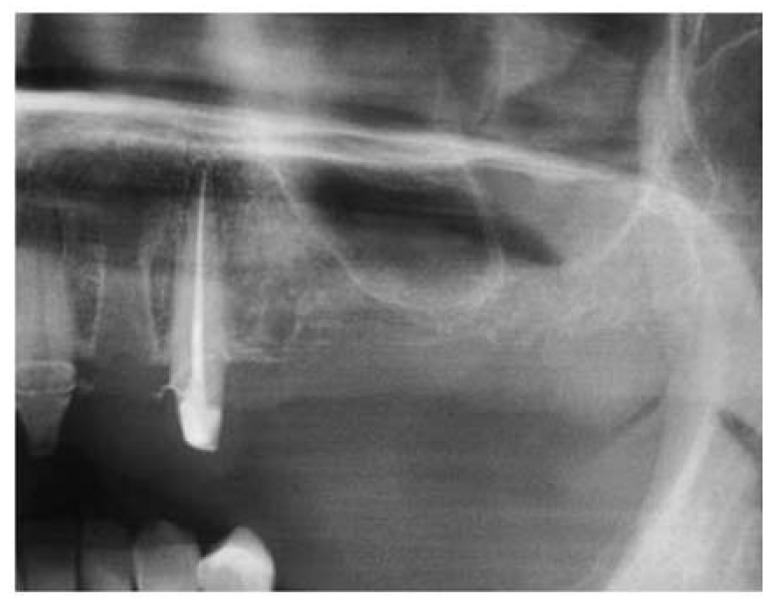
Preoperative panoramic X-ray view.

**Figure 2:**
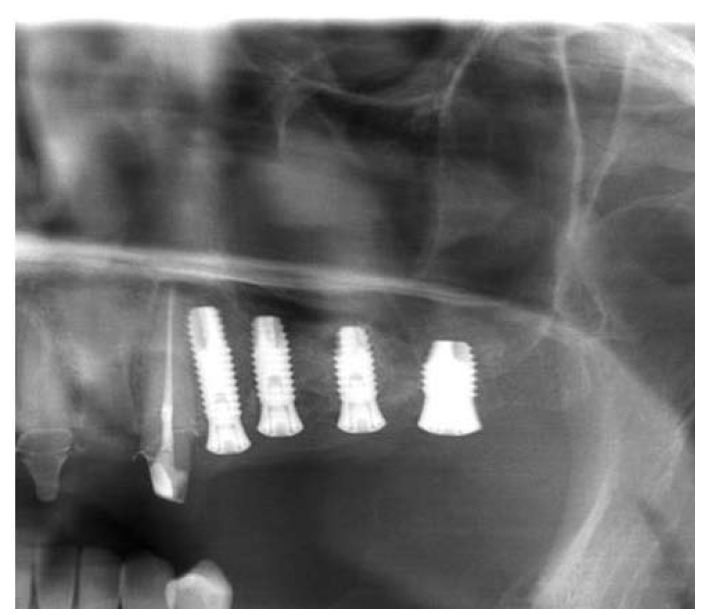
Postoperative panoramic X-ray view.

The bone gain achieved with sinus lift was determined from the postoperative panoramic X-rays. Distortion was calculated, taking as reference the length of the implant, to compensate for image amplification produced in the panoramic X-rays (Orthopantomografth OP100 R. Instrumentarium Imaging®, Tuusula, Finland). We traced the bone margin of the alveolar process of the upper maxilla and the floor of the maxillary sinus, measuring the distance between the two tracings at the level of the vertical axis of the implants – thus obtaining the radiological height of the alveolar crest (a). After bone grafting, the floor of the maxillary sinus was again traced, and measurement was made of the distance from the latter to the bone margin of the alveolar crest at the level of the vertical axis of the implants – this representing the radiological height of the alveolar crest after bone grafting (b). The difference between both recordings (b-a) yielded the corresponding bone gain (2), (Fig. 1,2).

All patients underwent radiological evaluation at implant placement and again 12 months after prosthetic loading. The images were calibrated with CliniView® (version 5.1, Instrumentarium Imaging®, Tuusula, Finland), and were subjected to quantitative measurements of bone loss around the implants. The bone height lost was determined from the difference between measurement at the time of fitting of the prosthesis and measurement 12 months after loading.

**Figure 3:**
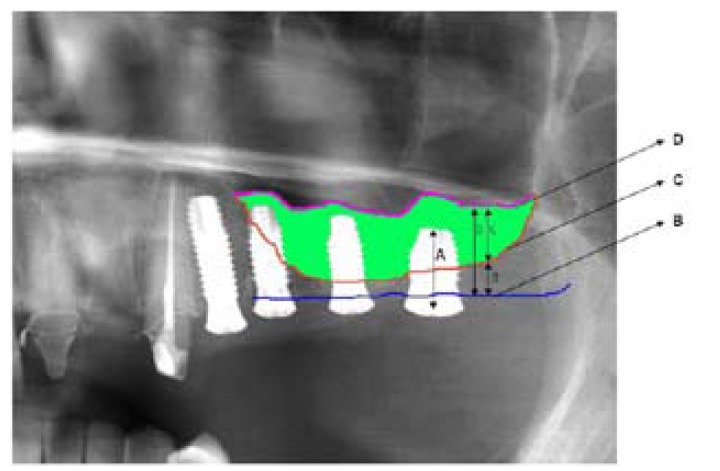
Postoperative panoramic X-ray view. Measurement of bone gain. A. Length of the implant. B. Occlusal line of the alveolar crest. C. Lower margin of the maxillary sinus before grafting. a. Radiological alveolar crest height before grafting. D. Lower margin of the maxillary sinus after grafting. b. Radiological alveolar crest height after grafting. Note the increase in height at the level of each of the implants. c. The bone gain (in green) is obtained by subtracting the radiological alveolar crest height before grafting from the height after grafting (c=b-a).

**Figure 4:**
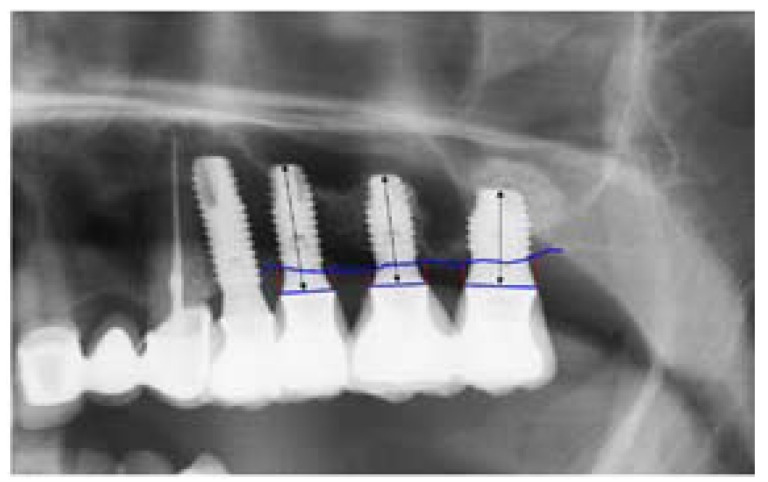
Postoperative panoramic X-ray view. Measurement of bone loss after prosthetic loading.

For measurement, we established two reference points at the junction between the implant and the prosthetic restoration, one located mesial and the other distal. These points are visible and locatable on all panoramic X-rays. A straight line was traced joining the two reference points, taking this axis to represent height zero. For the determination of bone loss, a perpendicular line was traced mesial and distal to the implant from the mentioned axis to the most corneal positioned bone (Fig. 3). Bone loss was obtained from the difference between the radiological alveolar crest height on the postoperative panoramic X-ray at prosthetic loading and the control panoramic X-ray obtained 12 months after loading. The difference between the values obtained at both measurement time points was used to represent bone loss mesial and distal to the implant the greater of the two being taken as the reference value (10,11) (Fig. 4).

A descriptive analysis was made of the study variables, with their corresponding frequency distributions and measures of central tendency and dispersion. Statistical comparisons between the two groups (rotary instrumentation and ultrasound) were made using the chi-squared test and Student t-test. The SPSS version 15.0 statistical package for Microsoft Windows was used throughout (SPSS Inc., Chicago, IL, USA). Statistical significance was considered for p<0.05.

## Results

Three patients were removed from the study (2 patients in which the study protocol was incomplete, and one case in which follow-up could not be carried out). A total of 45 patients were included, with a mean age of 50.2 years (range 29-69 years). There were 15 males and 30 females. A total of 57 sinus lift procedures were carried out (32 with the rotary technique and 25 with ultrasound). A total of 100 implants were placed. In 37 cases single-step surgery was used, i.e., sinus lift with simultaneous implant placement, while in 20 cases two-step surgery was used.

There were four perforations of Schneider’s membrane with the rotary technique (1 perforation measured 2 mm in diameter, 2 measured 5 mm, and 1 measured 6 mm in diameter) and a single perforation with the ultrasound technique (measuring 3 mm in diameter). In all cases of perforation of the sinus membrane we placed a reabsorb able collagen membrane (Lyostypt®, B. Braun, Aesculap, Tuttlingen, Germany) in contact with the sinus membrane, in the zone corresponding to the perforation.

Regarding the type of bone graft employed, particulate autologous bone was used in 40 implants, bovine heterologous bone (Bio-Oss®) in 34 cases, and combined particulate autologous bone and Bio-Oss® in 26 cases.

Forty-two implants were placed by conventional drilling, according to their length and diameter. In the remaining 58 implants we combined conventional drilling with the use of osteotomes.

Forty-five implants were submerged, requiring second-step surgery to replace the caps and leave them exposed, while 55 implants were placed initially exposed. A total of 53 Defcon® implants and 47 Straumann® implants were positioned. In 23 elevations no coating membrane was used, while in 34 a reabsorb able collagen membrane was applied.

Regarding alveolar crest atrophy, in 52 implants the radiological crest height before grafting was under 4 mm, in 38 implants the height was between 4 and 6 mm, and in 10 implants the crest height was over 8 mm. The maxillary atrophy observed on the extra oral panoramic X-rays proved bilateral in 12 cases and unilateral in 33 cases (12 on the right side and 21 on the left). ( Table 1) shows the mean radiological alveolar crest height before and after grafting, and the bone gain and loss, associated with the two techniques ( Table 1).

**Table 1:**
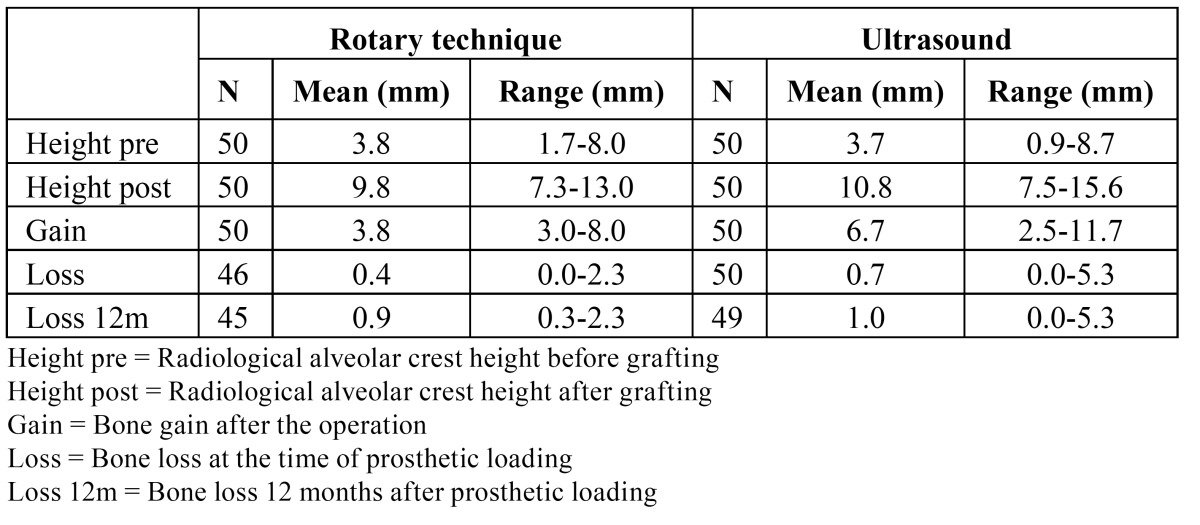
Mean previous bone height, mean millimeters gained after the operation, mean bone loss at the time of loading, and mean bone loss 12 months after loading, with the rotary technique and with ultrasound.

Five implants placed through sinus lift with the rotary technique failed before loading. Twelve months after loading no further implants were seen to have failed in this group, while a single implant failed in the ultrasound group. The implant success rate 12 months after loading was 90% with the rotary technique and 98% with ultrasound ( Table 2).

**Table 2:**
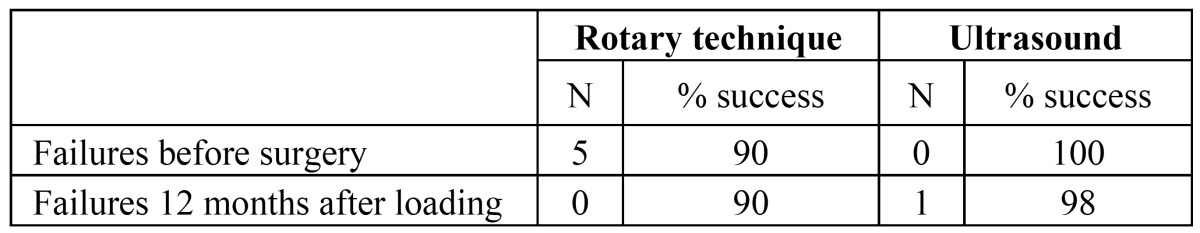
Number of failed implants and percentage success.

## Discussion

Of the 57 direct maxillary sinus lifts performed, a larger number of perforations of Schneider’s membrane was documented with the rotary technique than with the ultrasound technique. Thor et al. (12) in 20 patients, performed 27 direct maxillary sinus lift procedures with the rotary technique. There were 11 membrane perforations (41% of the overall operations). Using the same technique, Swartz-Arad et al. (3) obtained a similar result, with 36 perforations in 81 maxillary sinus lift procedures (44% of the operations). Ultrasound is associated with fewer perforations of the membrane. In this context, Vercellotti et al. (4) performed 21 direct maxillary sinus lift procedures in 15 patients, with a perforation rate of only 5%. In our series perforations were likewise less common with ultrasound (1.7% versus 7% with the rotary technique). In contrast, Barone et al. (8) treated 26 patients, 13 with the conventional rotary technique and 13 with ultrasound, and observed no statistically significant differences between the two techniques in terms of membrane perforation.

In all cases of perforation of the sinus membrane we placed a reabsorb able collagen membrane, as indicated by Hernandez-Alfaro et al. (13) for perforations measuring less than 5 mm in size. The bone grafts used in our study consisted of autologous bone, Bio-Oss®, or a 50% mixture of both.

In the present study, sinus lift with the ultrasound technique afforded a higher success rate than the rotary technique (98% versus 90%). However, Thor et al. (12) using the rotary technique, reported a success rate similar to that obtained in our series with the ultrasound technique.

Regarding bone gain, Vercellotti et al. (4) compared the bone regeneration achieved with the ultrasound technique versus the rotary technique. To this effect the authors performed a series of ostectomies with the two techniques, in one same dog. After 56 days of follow-up, greater bone regeneration was noted in the operations performed with ultrasound. In our study, the bone gain achieved after the operation and 12 months after loading was greater with the ultrasound technique (6.7 mm versus 5.9 mm with the rotary technique). However, Thor et al. (12) reported a mean bone gain of 6.5 mm with the rotary technique – this being very similar to the results obtained in our series with ultrasound. Some authors have also reported a greater bone volume when covering is carried out with a reabsorb able collagen membrane (14).

## Conclusions

Perforations of the sinus membrane are more frequent in direct sinus lift when using the rotary technique (7%) than with ultrasound (1.7%), and survival of the implants and the bone gain are greater with the ultrasound technique.

## References

[1] Sorní M, Guarinós J, García O, Peñarrocha M (2005 ). Implant rehabilitation of the atrophic upper jaw: a review of the literature since 1999. Med Oral Patol Oral Cir Bucal.

[2] Shlomi B, Horowitz I, Kahn A, Dobriyan A, Chaushu G (2004). The effect of sinus membrane perforation and repair with Lambone on the outcome of maxillary sinus floor augmentation: a radiographic assessment. Int J Oral Maxillofac Implants.

[3] Schwartz-Arad D, Herzberg R, Dolev E (2004). The prevalence of surgical complicationsof the sinus graft procedure and their impact on implant survival. J Periodontol.

[4] Vercellotti T, De Paoli S, Nevins M (2001). The piezoelectric bony window osteotomy and sinus membrane elevation: introduction of a new technique for simplification of the sinus augmentation procedure. Int J Periodontics Restorative Dent.

[5] Vercellotti T, Nevins ML, Kim DM, Nevins M, Wada K, Schenk RK (2005). Osseous response following respective therapy with piezosurgery. Int J Periodontics Restorative Dent.

[6] Torrella F, Pitarch J, Cabanes G, Anitua E (1998). Ultrasonic ostectomy for the surgical approach of the maxillary sinus: a technical note. Int J Oral Maxillofac Implants.

[7] Stübinger S, Kuttenberger J, Filippi A, Sader R, Zeilhofer HF (2005). Intraoral piezosurgery: preliminary results of a new technique. J Oral Maxillofac Surg.

[8] Barone A, Santini S, Sbordone L, Crespi R, Covani U (2006). A clinical study of the outcomes and complications associated with maxillary sinus augmentation. Int J Oral Maxillofac Implants.

[9] Buser D, Mericske-stern R, Dula K, Lang NP (1999). Clinical experience with one stage, non-submerged dental implants. Adv Dent Res.

[10] Boronat A, Peñarrocha M, Carrillo C, Marti E (2008). Marginal bone loss in dental implants subjected to early loading (6 to 8 weeks postplacement) with a retrospective short-term follow-up. J Oral Maxillofac Surg.

[11] Sánchez-Recio C, Peñarrocha-Diago M, Peñarrocha-Diago M, Peñarrocha-Oltra D (2010 ). Maxillary sinus lift performed using ultrasound. Evaluation of 21 patients. Med Oral Patol Oral Cir Bucal.

[12] Thor A, Sennerby L, Hirsch JM, Rasmusson L (2007). Bone formation at the maxillary sinus floor following simultaneous elevation of the mucosal lining and implant installation without graft material: an evaluation of 20 patients treated with 44 Astra Tech implants. J Oral Maxillofac Surg.

[13] Hernández-Alfaro F, Torradeflot MM, Marti C (2008). Prevalence and management of Schneiderian membrane perforations during sinuslift procedures. Clin Oral Implants Res.

[14] Froum SJ, Tarnow DP, Wallace SS, Rohrer MD, Cho SC (1998). Sinus floor elevation using anorganic bovine bone matrix (OsteoGraf/N) with and without autogenous bone: a clinical, histologic, radiographic, and histomorphometric analysis--Part 2 of an ongoing prospective study. Int J Periodontics Restorative Dent.

